# Individual-based socioeconomic vulnerability and deprivation indices: a scoping review

**DOI:** 10.3389/fpubh.2024.1403723

**Published:** 2024-08-14

**Authors:** Dionysios Palermos, Elpida Pavi, Panagiotis Halvatsiotis, Polyxeni Mangoulia, Theodoros N. Sergentanis, Theodora Psaltopoulou

**Affiliations:** ^1^Department of Public Health Policy, School of Public Health, University of West Attica, Athens, Greece; ^2^Second Propaedeutic Department of Internal Medicine, School of Medicine, “Attikon” University Hospital, National and Kapodistrian University of Athens, Athens, Greece; ^3^Department of Nursing, School of Health Sciences, National and Kapodistrian University of Athens, Athens, Greece; ^4^Department of Clinical Therapeutics, “Alexandra” Hospital, School of Medicine, National and Kapodistrian University of Athens, Athens, Greece

**Keywords:** socioeconomic status, health inequalities, deprivation, socioeconomic vulnerability, index

## Abstract

Several individual-based social deprivation and vulnerability indices have been developed to measure the negative impact of low socioeconomic status on health outcomes. However, their variables and measurable characteristics have not been unequivocally assessed. A comprehensive database literature scoping review was performed to identify all individual-based social deprivation and vulnerability indices. Area-based indices and those developed for pediatric populations were excluded. Data were extracted from all eligible studies and their methodology was assessed with quality criteria. A total of 14 indices were identified, of which 64% (9/14) measured social deprivation and 36% (5/14) measured socioeconomic vulnerability. Sum of weights was the most common scoring system, present in 43% (6/14) of all indices, with no exclusive domains to either vulnerability or deprivation indices. A total of 83 different variables were identified; a very frequent variable (29%; 5/14) related to an individual’s social relationships was “seen any family or friends or neighbors.” Only five deprivation indices reported a specific internal consistency measure, while no indices reported data on reproducibility. This is the first scoping review of individual-based deprivation and vulnerability indices, which may be used interchangeably when measuring the impact of SES on health outcomes.

## 1 Introduction

The negative impact of low socioeconomic status (SES) on several diseases has been well established (1–3), making the narrowing of social inequalities and poverty reduction top priorities for both public health researchers and policy makers (4). Socioeconomic vulnerability, broadly defined as the inability of certain individuals to cope with adverse shocks (e.g., natural disasters, economic crises, etc.) (5), has been measured with individual (6) or area-based (geographic) indices (7, 8), using questionnaires to obtain information about specific indicators, such as financial stability, education, employment, housing conditions, and personal relationships. These indices have been utilized in epidemiological studies to detect possible causal relationships between SES and various health outcomes (9–13). Similarly, the concept of deprivation, defined as a lack of resources to maintain a socially acceptable lifestyle (14), has been developed to assess socioeconomic position in adults and children (15), by portraying actual living conditions through the use of questionnaire-based measures, at the individual (16, 17) and geographic level (18–21). Material deprivation can be measured with indicators relative to nutrition, clothing, housing, and employment, whereas social deprivation is more challenging to assess and refers to the conditions leading to social exclusion (22). A plethora of epidemiological studies have revealed the association of deprivation with deteriorated health outcomes (23–25).

Although several indices of individual socioeconomical vulnerability and deprivation have been utilized in public health and clinical research, the determination of widely acceptable living conditions is dependent on several temporal and geographic factors (26). Taking also into consideration the significant variability in study design and the ambiguity of measurement properties of these indices, a particularly heterogenous landscape is created. While these two concepts have clearly distinct definitions, significant differences regarding their measurable characteristics and their individual variables still remain unclear. Therefore, it is necessary to identify all relevant indices and examine the different types of variables of the respective questionnaires, in order to organize data pertaining to the approach utilized in constructing and utilizing deprivation indices in research and practical applications. In this context, the present scoping review aims to critically assess the methodological aspects of individual-based socioeconomic vulnerability and deprivation indices, focusing only on those developed for adult participants.

## 2 Materials and methods

This scoping review was performed according to the Preferred Reporting Items for Systematic Reviews and Meta-Analysis Protocols extension for scoping reviews (PRISMA-ScR) (27). The PRISMA-ScR Checklist is provided as a Supplementary Table S1.

### 2.1 Data source and search strategy

A comprehensive literature search was conducted in MEDLINE (PUBMED) and SCOPUS on March 31, 2023, using the following algorithm: “((Individual) OR (individual-based) AND ((social vulnerability) OR (socioeconomic vulnerability)) OR deprivation OR (material deprivation) OR (social deprivation) AND index).” We also used the queries “individual socioeconomic vulnerability index” and “individual deprivation index” for a Google search. Additionally, we performed the snowball technique (reference screening for eligible studies) on reviews, systematic reviews and/or meta-analyses found in the aforementioned literature search.

### 2.2 Study selection

Only individual-based indices were considered eligible for this scoping review, with data derived from questionnaires. Since vulnerability and deprivation seem to affect the responders but also their families, we also included indices with variables examining socioeconomic characteristics at the household level. Eligible studies had to describe the methodology with which socioeconomic vulnerability or deprivation indices were developed. Area-based and geographic indices deriving ecological data or data from censuses were excluded from this scoping review. Indices developed for children were also excluded due to their different context with respect to study design, variables and outcomes. No language restrictions were applied during the study selection process.

### 2.3 Data extraction and synthesis

Two authors (DP and TS) independently screened the title, abstract, and full text of the resulting articles and extracted the data. A designated third reviewer (TP) examined the data and resolved any discrepancy that occurred through mutual consultation. The following data were extracted (if available) from all eligible studies: Index title, country of study conduct, scoring system, study sample, outcome measures, types of variables, statistical methodology, strengths, and limitations. All data were recorded in Word and presented descriptively in tables and figures. With no specific quality criteria in effect for deprivation or vulnerability indices, we decided to use the criteria proposed by Terwee et al. (28) to characterize the quality of measurement properties of all indices. A similar approach has been used by Fouchard et al. (29) to characterize the respective properties of deprivation indices. Based on these criteria, we rated each individual property of every index as positive, negative, or indeterminate, according to the data provided in the publication. Since these criteria were created for health status questionnaires, a number of differences were expected to arise when assessing deprivation and vulnerability indices; we chose to modify the “floor or ceiling effect” and to assess only the upper limit of <15% of the respondents achieving the highest possible index scores (most deprived/vulnerable), on the basis that most individuals are expected to not be deprived or vulnerable.

## 3 Results

**Figure 1:**
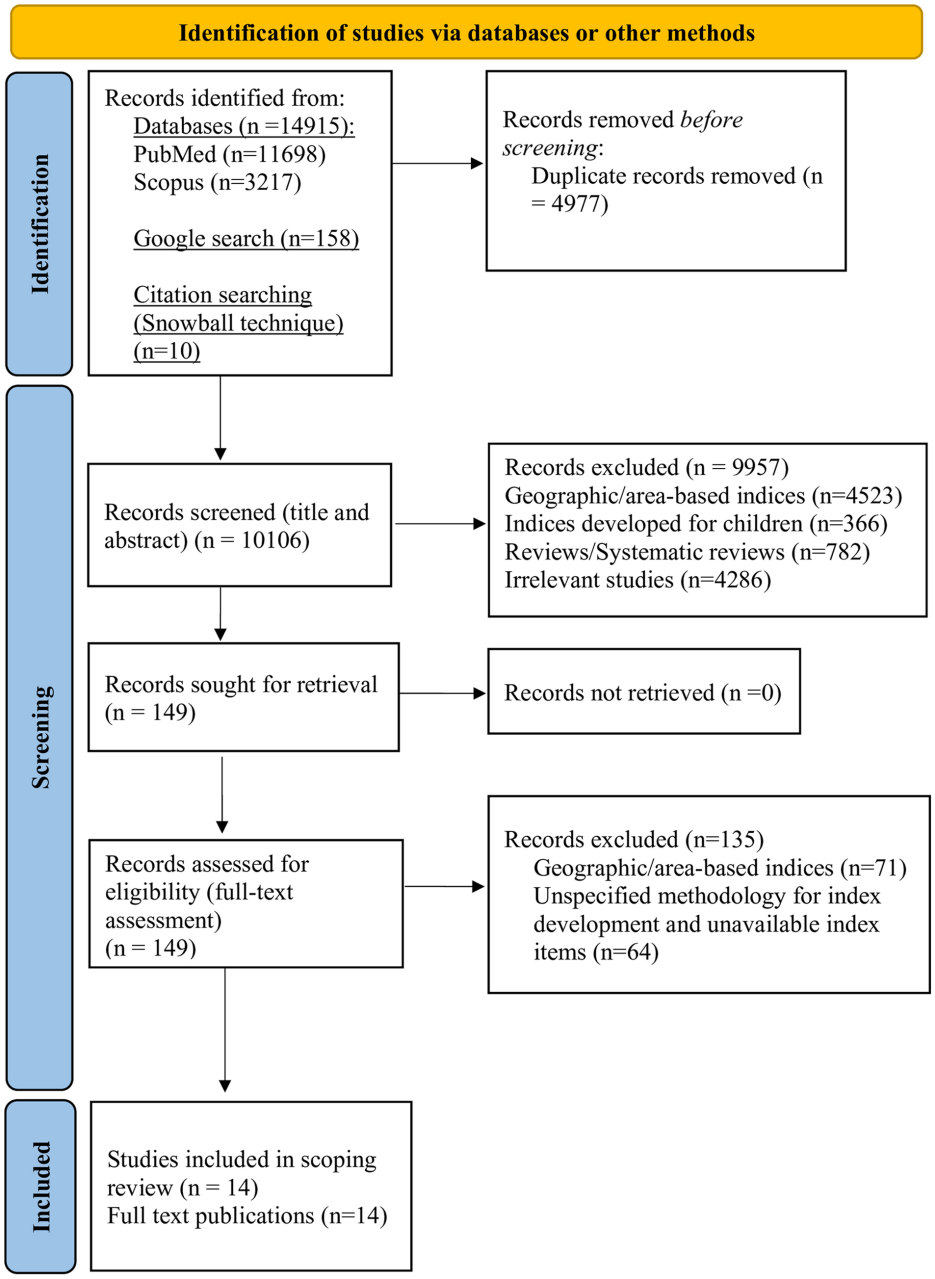
PRISMA flow chart for study selection. Adapted from Page et al. (30), licensed under CC BY 4.0.

The detailed study selection process is presented in the flow chart diagram (Figure 1). A total of 15,083 records were identified, with 4,977 excluded as duplicates, and the remaining 10,106 were screened (title and abstract); of those, 4,523 were excluded because they pertained to geographic/area-based indices, 366 because they were developed for children, 782 due to being reviews or systematic reviews, and 4,286 due to being irrelevant. Thus, we ended up with 149 articles, for which full-text examination was performed. We then excluded 135 records; 71 due to being geographic/area-based indices and 64 because they did not specify index items and development methodology. Finally, we identified a total of 14 eligible indices, of which 64.3% (9/14) (16, 17, 26, 31–36) were measuring social deprivation and 35.7% (5/14) (12, 37–40) were measuring socioeconomic vulnerability. One study (31) was referring to “social handicap” instead of deprivation, with no significant conceptual differences between the two terms, thus we decided to include it in the deprivation category to facilitate categorization. This questionnaire reported 111 items; however, it described in detail six domains of social handicap and only 18 distinct variables, which we extracted for our research. The main characteristics of all indices are presented in Tables 1A,B, with their variables categorized according to the most frequent domains, namely:

- Financial situation
- Insurance (Social security)
- Employment
- Education
- Basic living standards (quality of housing and material comfort)
- Receipt of social benefit (from government or local community or other organization)
- Family and relationships
- Health (mental or physical)
- Leisure (available time and resources for hobbies or other similar activities)

**Table 1:** Characteristics of individual-based deprivation and socioeconomic vulnerability indices.

A							
Index title	NZiDep (16)	DiPCare-Q (17)	PRECAR (26)	EPICES (34)	ODI (35)	MSD (32)	Handicap social (30)
Country	New Zealand	Switzerland	France	France	Canada	EU (27 countries)	France
No. of variables	8	16	14	11	10	13	18
Scoring system	Sum of “yes” answers	Weighted sum of three subindices	Sum of points (range: 0–27)	Sum of weights of positive answers (range: 0–100)	List of 10 items	List of 13 items	Sum of weights of answers
Deprivation/Vulnerability threshold(s)	Five categories according to number of YES answers: 0,1,2,3–4,5+	Continuous	Index = 10 isolates 20% of most deprived people	Socially deprived: Score > 30,17	Lacking ≥2 items	MSD: Lacking ≥5 items, SMSD: Lacking ≥7 items	Class 1: absence of social handicap; Class 2: moderate handicap; Class 3: strong handicap.
Household variables	No	Yes (*n* = 5)	No	No	No	Yes (*n* = 7)	No
No. of variables pertaining to:							
Financial situation	-	3	1	-	-	2	2
Insurance	-	-	1	1	-	-	-
Employment	1	-	2	-	-	-	2
Education	-	-	1	-	-	-	1
Basic living standards	4	5	2	2	6	8	6
Receipt of social benefit	3	-	-	1	-	-	2
Family and relationships	-	2	5	4	1	1	2
Health (mental or physical)	-	4	1	-	1	-	3
Leisure	-	2	-	3	1	2	-
Other	-	-	1	-	1	-	-

B							
Index title	FWID (31)	SDI (33)	SVI (36)	SEV (12)	rSVI (38)	SEVI (37)	Score de Pascal (39)
Country	Turkey	United States	Canada	Korea	Denmark	China	France
No. of variables	23	8	32	6	10	6	5
Scoring system	Sum of weights of positive answers	Sum of weighted items	Sum of deficit scores divided by no. of items (range 0–1)	Sum of points of answers (range 0–6)	Sum of weighted items	Sum of six values divided by 6 (range 0–1)	List of five items
Deprivation threshold(s)	NR	Continuous	Socially vulnerable: SVI > 0.37	Continuous	Socially vulnerable: rSVI≥5	Continuous	Deprived if YES to questions 1, 3, or 4 or NO to question 2
Household variables	Yes (*n* = 13)	No	No	No	Yes	No	No
No. of variables pertaining to:							
Financial situation	4	2	1	2	1	2	-
Insurance	2	1	-	1	-	-	2
Employment	1	1	-	-	1	1	1
Education	1	1	4	1	1	1	-
Basic living standards	13	2	3	-	-	-	-
Receipt of social benefit	-		-	-	-	-	1
Family and relationships	-		5	-	1	-	-
Health (mental or physical)	2		14	-	4	1	1
Leisure	-		2	1	-	-	-
Other	-		3	1	2	1	-

NZiDep, New Zealand index of deprivation; DiPCare-Q, Deprivation in primary care questionnaire; EPICES, Evaluation de la Precarite et des Inegalites de sante dans les Centers d’Examens de Sante; ODI, Ontario Deprivation Index; MSD, Material and social deprivation; SMSD, Severe material and social deprivation; European Union. FWID, Factor weighted index of deprivation; SDI, Social deprivation index; SVI; Social vulnerability index; SEV(I), Socioeconomic vulnerability index; rSVI, register-based social vulnerability index; NR, Not reported.

Among all evaluable indices the median [interquartile range (IQR)] number of variables was 11.0 (8.0–16.0), with only two distinct outliers (36, 37) which had 32 and 23 variables, respectively.

### 3.1 Index scoring systems

We observed four distinct scoring systems.

#### 3.1.1 Sum of weights

This was the most common scoring system, present in 55.6% (5/9) (17, 31, 32, 34, 35) and 20.0% (1/5) (39) of the deprivation and vulnerability indices, respectively. In these cases, researchers assigned specific weights (points) to each item, therefore the most weighted answers played a more significant role in shaping the total score, with higher scores denoting more deprived or vulnerable individuals. Choice of weight was based on the prevalence of each item in the population (35), the subjective perceptions of necessity (32), or the researchers’ experience on social inequality (31, 39). For example, in the Evaluation of Deprivation and Inequalities in Health Examination Centers (EPICES) index (35), the “Do you live as a couple?” item had a weight of −8.28, indicating that individuals who do not live alone are less prone to deprivation; on the contrary, the “Do you sometimes meet with a social worker (welfare worker, educator)?” item had a weight of 10.06, indicating that people who meet with a social worker are more prone to deprivation. All indices using this method had only binary questions that could be answered with YES/NO. Most such indices did not define a threshold for determining deprived or vulnerable individuals, with only one index (39) utilizing a predetermined value for this purpose.

#### 3.1.2 Sum of points

This scoring system was found in 60.0% (3/5) of the vulnerability indices (12, 37, 38) and only in one (11.1%; 1/9) deprivation index (26). This method was used for indices having questions with more than two possible answers, in which increasing values were assigned depending on the extent of vulnerability/deprivation. For example, in the PRECAR index (26), the question “Generally speaking, would you say that you feel?” has the following four possible answers, with the assigned points increasing proportionally to the extent of loneliness: Very surrounded (0 points), somewhat surrounded (1 point), rather lonely (2 points), and very lonely (3 points).

#### 3.1.3 List of items

This scoring method was present in two deprivation indices (22.2%; 2/9) (33, 36) and one vulnerability index (20.0%; 1/5) (40), in which a list of specific deprivation-related items was included. If participants lacked a certain number of items, they were categorized as materially and/or socially deprived. For example, participants completing the EU Material and Social Deprivation (MSD) 13-item questionnaire (33) were considered materially and socially deprived if they lacked at least five items and severely materially and socially deprived if they lack at least seven items.

#### 3.1.4 Sum of positive answers

Only one deprivation index (11.1%; 1/9) (16) utilized this scoring system, in which the total score was calculated by summing the “yes” answers to the eight deprivation questions. This method was used only for binomial questions, while five categories of deprivation were determined according to the number of positive answers.

### 3.2 Domains and variables of deprivation and vulnerability

We observed that there were no exclusive domains to either vulnerability or deprivation indices (Tables 1A,B); the most frequent domain was “financial situation,” present in 71.4% (10/14) of all indices and specifically in 80.0% (4/5) of the vulnerability indices. The domain “basic living standards” was present in all deprivation indices (100.0%; 9/9) however it was rather uncommon in vulnerability indices, found in only one such index (20.0%; 1/5). “Education” on the other hand was the only domain that was present in most vulnerability indices (80.0%; 4/5) but in less than half of the deprivation indices (44.4%; 4/9). A total of 83 different variables were identified. All variables that were found in ≥2 indices are presented in Table 2 in descending frequency order, while a list of variables found in only one index are presented in Supplementary Table S2.

**Table 2:** Frequency of variables of individual-based deprivation and vulnerability indices.

Frequency	Variable
Six times	Educational status
	Income (household or individual)
	Insurance (social security, etc.)
Five times	Employment status (employed, unemployed, etc.)
	Seen any family members or friends or neighbors
	Housing tenure (owning, renting, etc.)
Four times	Emotional/marital status
	Perception of financial status
	Wealth or savings (family or individual)
Three times	Professional category (type of employment)
	Physical handicap
	Able to afford meat, fish or vegetarian equivalent at least every other day
	Having a hobby or leisure activity
	Cannot keep the home adequately warm in winter
	Financial/material help in need from someone close to the person
	Being on a means-tested benefit (government aid based on income)
	Gone on holiday (or ability to afford holiday)
	Addiction
	Housing condition
Two times	Nationality
	Living arrangement (living alone, etc.)
	Scared of losing housing
	Cannot afford new clothes
	Cannot afford new furniture
	Cannot afford to have two pairs of properly fitting shoes
	Help to get food (special food grants or food banks)
	Doing without fresh fruit and vegetables
	Afford to attend shows (cinema, theater, etc.)
	Afford to participate in sports activities
	Difficulties reimbursing loan(s)
	Difficulties paying bills
	Limited access to healthcare
	Psychic handicap
	Cannot afford to spend a small amount of money each week on him/herself (“pocket money”)
	No access to the internet at all
	Feelings of loneliness
	Able to get around the community
	Job health and safety
	Sufficient supply of pharmacy/medical care/grocery in reasonable distance
	Cannot afford a car

The most frequent variables were “educational status,” “insurance,” and “income (household or individual)” [in six indices each (42.9%; 6/14)]. The questions pertaining to “educational status” mostly had ≥3 answers, with lowest education assigned to the most points. Only one index (34) had a dichotomous question with two possible answers: “0–17 years of education” vs. “17 + years of education.” This variable was present only in two deprivation indices (26, 34), as opposed to 80.0% (4/5) of vulnerability indices (12, 37–39). With respect to “income,” participants were most commonly assigned to ≥2 categories, depending on a country-specific financial amount, while two indices (32, 34) allowed a continuous estimation of income, with the Turkish index developed by Eroglu et al. (29) formulating the question as “Real disposable monthly household income, i.e., income – (rent+ fixed travel expenses).” This was the most balanced variable being present in three deprivation (26, 32, 34) and three vulnerability indices (12, 37, 39). “Insurance” was the most heterogenous variable, as a number of country-specific variations were identified since social security systems vary extensively among different countries; a clear distinction was made for individuals having private or public insurance, or no insurance at all. This variable was included in four deprivation indices (26, 32, 34, 35) and two vulnerability indices (12, 40).

Variables that were found in five different indices (35.7%; 5/14) were “employment status,” “seen any family members or friends or neighbors,” and “housing tenure (owning, renting, etc.).” “Employment status” was present in three deprivation (16, 26, 34) and two vulnerability indices (39, 40), and was most commonly measured dichotomously with two possible answers, depending on the respondents’ active employment or receipt of unemployment benefits, with only one index (26) having the following five distinct categories of increasing points: employed (0 points), student (0 points), retired (1 point), inactive (1 point), and unemployed (2 points). The “seen any family members or friends or neighbors” variable was included only in deprivation indices (17, 31, 33, 35, 36), and was formulated most commonly as the following dichotomous question: “Do you spend time with friends or family over for a meal at least once a month/twice per year?” “Housing tenure” was another variable found only in deprivation indices (26, 31, 32, 34, 35), examining whether respondents were owning or renting their residence.

Among variables that were identified four times in all indices (28.6%; 4/14) “emotional/marital status” was included in two deprivation (26, 35) and two vulnerability indices (37, 39). Married respondents or those in a relationship received the lowest score, whereas divorced, widowed or never married individuals received the highest score in these questions. “Perception of financial status” was a rather heterogenous variable, found in three deprivation (26, 31, 35) and one vulnerability index (12); this variable was utilized to measure either the economic satisfaction or the extent of financial difficulties as perceived by the individual. Finally, “wealth or savings (family or individual)” was also present in three deprivation (31, 32, 34) and one vulnerability index (38).

### 3.3 Measurement properties of deprivation and vulnerability indices

Finally, we sought to review the individual methodological characteristics of the aforementioned indices in order to determine which are the most methodologically sound. To achieve this, we used the quality criteria proposed by Terwee et al. (28) to assess their properties (Table 3). An overview of internal consistency measures for all indices is presented in Table 4. We observed that only five deprivation indices reported a specific internal consistency measure, with this information completely lacking from vulnerability indices.

**Table 3:** Measurement properties of individual-based deprivation and vulnerability indices.

Index	Content validity	Internal consistency	Construct validity	Reproducibility		Responsiveness	Floor or ceiling effect	Interpretability
				Agreement	Reliability			
NZiDep	+	+	?	0	0	0	+	0
DiPCare-Q	−	+	?	0	+	0	0	0
PRECAR	+	−	+	0	0	0	+	+
EPICES	−	−	+	0	0	0	0	+
ODI	+	?	0	0	0	0	?	?
MSD	+	+	?	0	0	0	+	+
Handicap social	−	0	0	0	0	0	0	+
FWID	+	?	?	0	0	?	?	0
SDI	−	?	?	0	0	0	+	?
SVI	+	?	0	0	0	0	+	0
SEV	?	?	?	0	0	0	−	0
rSVI	−	?	+	0	0	0	+	0
SEVI	−	0	0	0	0	0	−	0
Score de Pascal	−	+	+	0	0	0	−	0

+, Positive rating; ?, Indeterminate rating, −, Negative rating; 0, No information available.

NZiDep, New Zealand index of deprivation; DiPCare-Q, Deprivation in primary care questionnaire; EPICES, Evaluation de la Precarite et des Inegalites de sante dans les Centers d’Examens de Sante; ODI, Ontario deprivation index; MSD, Material and social deprivation; SMSD, Severe material and social deprivation; European Union, FWID, Factor weighted index of deprivation; SDI, Social deprivation index; SVI; Social vulnerability index; SEV(I), Socioeconomic vulnerability index; rSVI, Register-based social vulnerability index; NR, Not reported.

**Table 4:** Overview of internal consistency measures of all deprivation and vulnerability indices.

Index	Internal consistency measure
NZiDep	Cronbach’s alpha = 0.816
DiPCare-Q	KR20^d^ = 0.827, KR20^v^ = 0.778
PRECAR	Cronbach’s alpha = 0.680
EPICES	Cronbach’s alpha = 0.410
ODI	NR
MSD	Cronbach’s alpha = 0.85 (range: 0.76–89 according to individual country)
Handicap social	NR
FWID	NR
SDI	NR
SVI	NR
SEV	NR
rSVI	NR
SEVI	NR
Score de Pascal	NR

KR20^d^, Kuder–Richardson Formula 20 for derivation set; KR20^v^, Kuder–Richardson Formula 20 for validation set.

NZiDep, New Zealand index of deprivation; DiPCare-Q, Deprivation in primary care questionnaire; EPICES, Evaluation de la Precarite et des Inegalites de sante dans les Centers d’Examens de Sante; ODI, Ontario deprivation index; MSD, Material and social deprivation; SMSD, Severe material and social deprivation; European Union, FWID, Factor weighted index of deprivation; SDI, Social deprivation index; SVI; Social vulnerability index; SEV(I), Socioeconomic vulnerability index; rSVI, Register-based social vulnerability index; NR, Not Reported.

## 4 Discussion

In this scoping review, a total of 14 different indices measuring both socioeconomic deprivation and vulnerability were identified. The first takeaway message from this process was that no significant differences with regard to study design or variables were found between deprivation and vulnerability indices, with no exclusive scoring system or domain. The scoring methods most commonly utilized were “sum of weights” for deprivation indices and “sum of points” for vulnerability indices, whereas “basic living standards” was the most common domain in deprivation indices, as opposed to “education,” which was very frequent in vulnerability indices. There were also no exclusive variables for any type of index, however “income” and “seen any family members or friends or neighbors” were common in deprivation indices, whereas “educational status” was most commonly found in vulnerability indices. Therefore, deprivation and vulnerability indices may be used interchangeably when trying to measure the impact of SES on various health outcomes, especially considering the conceptual relationship between the two concepts.

Social deprivation and social vulnerability often intersect and mutually reinforce each other; it is expected that materially and socially deprived communities may lack the necessary resources to prepare for and respond to adverse shocks, making them more vulnerable to harm. Similarly, socioeconomically vulnerable individuals may be more prone to social deprivation, owing to their limited access to resources and support systems. Moreover, both deprived and vulnerable individuals face increasing social exclusion due to their hindered SES (41), which has been correlated with health inequalities and adverse outcomes (42, 43). In the future, researchers might benefit from utilizing and further improving already existing indices, by adding or removing certain items depending on several temporal and geographic factors. Thus, the assessment of vulnerability and deprivation could be more consistent and comparable across different countries and time periods.

Among the most frequent variables (found in ≥5 different indices), the majority were related to traditional indicators of socioeconomic status, such as income, education, and employment (44, 45). Interestingly, the only frequent variable closely related to an individual’s social relationships was “seen any family or friends or neighbors.” A substantial body of evidence has shown that healthy social relationships contribute to well-being and good health, through various psychological, behavioral, and physiological pathways (46–49). On the other hand, low-quality social ties have been linked with increased incidence of stress (50), cardiovascular disease (51), and mortality (52), even when controlling for other variables that might influence the aforementioned outcomes. Thus, this variable emerged as of utmost importance and should be included in such questionnaires, since it may detect the extent of social exclusion experienced by an individual.

Regarding their measurement properties, a considerable amount of missing data in regard to deprivation and vulnerability indices were expected as these criteria were proposed for assessing health status questionnaires, which ipso facto are required to provide extensive details about their design and methodology, while they are also required to be validated in a more rigorous manner (28). Indeed, we observed that almost all indices did not report any data on reproducibility, which refers to the degree to which repeated assessment in the same population provide similar responses (28). Only one deprivation index (17) had data on reliability, which refers to the degree to which participants may be distinguished from each other, measured by the interclass correlation coefficient (ICC). No indices provided any data on responsiveness, a measure of longitudinal validity broadly defined as the ability of an index to detect even small clinically important changes over time. In order to provide data on the aforementioned properties, studies should have been replicated in the same population, which obviously presents difficulties as they are non-interventional epidemiological studies, in which participant retention is particularly challenging (53).

Among all indices, a very well developed and validated index is the MSD index, which was developed across the 27 countries of the European Union (EU) in a rigorous manner, while its items are constantly being revised and optimized (33). Additionally, the NZiDep (16) and DiPCare-Q (17) are two well-developed and versatile deprivation indices, which have been used in several epidemiological studies (54–61), while the PRECAR index (26), developed in 2022, is also properly developed and validated, possessing robust measurement properties. Notably, the SVI (37) was the only vulnerability index utilized in several epidemiological studies (7, 13, 62–64).

To our knowledge, this is the first scoping review of individual-based deprivation and vulnerability indices. We believe that no relevant indices were missed, owing to the rigorous search strategy that was applied, that included a search algorithm, several search queries, and the snowball technique. The issue of missing data arose when we assessed the measurement properties of these indices, especially in highly technical criteria, such as reproducibility and responsiveness. Our study is limited by the exclusion of geographic-and area-based indices, such as the CDC/ATSDR Social Vulnerability Index (CDC/ATSDR SVI) (65); interestingly, a recent study in the United Kingdom highlighted the limitations of geographic/area-based indices in identifying individuals deprived at the employment or income level (66). Another limitation is the lack of registration of the protocol in an international database. However, we believe that in the absence of a consensus and taking into consideration the lack of data in this area, this assessment is very valuable as it reveals where greater effort needs to be made in reporting methodological characteristics, in order to improve the overall quality of these indices, while simultaneously guiding future index selection and application in clinical and public health research. Thus, studies aiming to determine the relationship between SES and various health outcomes by using individual-based deprivation or vulnerability indices may provide more methodologically sound results that will help to bridge the gap between research and public health policymaking.

## 5 Conclusion

In conclusion, this scoping review highlights the various individual-based indices, which can be used for measuring socioeconomic deprivation and vulnerability. It is evident that these indices have largely been used interchangeably to measure the impact of SES on health outcomes, as no domains or variables exclusive to either vulnerability or deprivation indices were observed. However, the review also reveals a considerable amount of missing data in regard to the measurement properties of these indices, especially in highly technical criteria such as reproducibility and responsiveness. This calls for greater effort in reporting methodological characteristics to improve the overall quality of these indices. The importance of including variables related to an individual’s social relationships is also emphasized, as they have been shown to have a significant impact on well-being and health outcomes. Overall, studies using individual-based deprivation or vulnerability indices can be used effectively to help bridge the gap between research and public health policy making.

## Data availability statement

The original contributions presented in the study are included in the article/Supplementary material, further inquiries can be directed to the corresponding author/s.

## Author contributions

DP: Conceptualization, Writing – original draft. EP: Writing – review & editing. PH: Writing – review & editing. PM: Writing – review & editing. TS: Writing – review & editing, Conceptualization. TP: Conceptualization, Writing – review & editing.

## References

[1] Adler NE Ostrove JM. Socioeconomic status and health: what we know and what we don't. Ann N Y Acad Sci. (1999) 896:3–15. doi: 10.1111/j.1749-6632.1999.tb08101.x 10681884

[2] Winkleby MA Jatulis DE Frank E Fortmann SP. Socioeconomic status and health: how education, income, and occupation contribute to risk factors for cardiovascular disease. Am J Public Health. (1992) 82:816–20. doi: 10.2105/AJPH.82.6.816, PMID: 1585961 PMC1694190

[3] Pathirana TI Jackson CA. Socioeconomic status and multimorbidity: a systematic review and meta-analysis. Aust N Z J Public Health. (2018) 42:186–94. doi: 10.1111/1753-6405.12762, PMID: 29442409

[4] Arntzen A Bøe T Dahl E Drange N Eikemo TA Elstad JI. 29 recommendations to combat social inequalities in health. The Norwegian council on social inequalities in health. Scand J Public Health. (2019) 47:598–605. doi: 10.1177/1403494819851364, PMID: 31512561

[5] Zimmermann A. Social Vulnerability as an Analytical Perspective, Berlin, Germany: Population Europe Secretariat (2017).

[6] Park E Ko Y. Socioeconomic vulnerability index and obesity among Korean adults. Int J Environ Res Public Health. (2021) 18:13370. doi: 10.3390/ijerph182413370, PMID: 34948979 PMC8704761

[7] Andrew MK Fisk JD Rockwood K. Social vulnerability and prefrontal cortical function in elderly people: a report from the Canadian study of health and aging. Int Psychogeriatr. (2011) 23:450–8. doi: 10.1017/S1041610210001195, PMID: 20699047

[8] Herrera-Escobar JP Uribe-Leitz T Wang J Orlas CP Moheb ME Lamarre TE. The social vulnerability index and long-term outcomes after traumatic injury. Ann Surg. (2022) 276:22–9. doi: 10.1097/SLA.0000000000005471 35703455

[9] Biggs EN Maloney PM Rung AL Peters ES Robinson WT. The relationship between social vulnerability and COVID-19 incidence among Louisiana census tracts. Front Public Health. (2020) 8:617976. doi: 10.3389/fpubh.2020.617976 33553098 PMC7856141

[10] Nguyen TN Ngangue P Bouhali T Ryan BL Stewart M Fortin M. Social vulnerability in patients with multimorbidity: a cross-sectional analysis. Int J Environ Res Public Health. (2019) 16:1244. doi: 10.3390/ijerph16071244, PMID: 30965571 PMC6480630

[11] Phelos HM Deeb AP Brown JB. Can social vulnerability indices predict county trauma fatality rates? J Trauma Acute Care Surg. (2021) 91:399–405. doi: 10.1097/TA.0000000000003228, PMID: 33852559 PMC8375410

[12] Sang Kyun P Youngsi H Byungtaek O Junyoung L Seungwan H Sunghi K. The relationship between socioeconomic vulnerability and cognitive impairment among aged people in Korea. Kor J Clin Geri. (2017) 18:74–81. doi: 10.15656/kjcg.2017.18.2.74

[13] Wallace LM Theou O Pena F Rockwood K Andrew MK. Social vulnerability as a predictor of mortality and disability: cross-country differences in the survey of health, aging, and retirement in Europe (SHARE). Aging Clin Exp Res. (2015) 27:365–72. doi: 10.1007/s40520-014-0271-6, PMID: 25213145

[14] Townsend P Phillimore P Beattie A. Health and deprivation. Nurs Stand. (1988) 2:34. doi: 10.7748/ns.2.17.34.s66 27415096

[15] Laporte R Babe P Jouve E Daguzan A Mazoue F Minodier P. Developing and validating an individual-level deprivation index for children&rsquo; s health in France. Int J Environ Res Public Health. (2022) 19:16949. doi: 10.3390/ijerph192416949, PMID: 36554830 PMC9816939

[16] Salmond C Crampton P King P Waldegrave C. NZiDep: a New Zealand index of socioeconomic deprivation for individuals. Soc Sci Med. (2006) 62:1474–85. doi: 10.1016/j.socscimed.2005.08.008 16154674

[17] Vaucher P Bischoff T Diserens EA Herzig L Meystre-Agustoni G Panese F. Detecting and measuring deprivation in primary care: development, reliability and validity of a self-reported questionnaire: the DiPCare-Q. BMJ Open. (2012) 2:e000692. doi: 10.1136/bmjopen-2011-000692, PMID: 22307103 PMC3274718

[18] Smith GD Whitley E Dorling D Gunnell D. Area based measures of social and economic circumstances: cause specific mortality patterns depend on the choice of index. J Epidemiol Community Health. (2001) 55:149–50. doi: 10.1136/jech.55.2.149, PMID: 11154256 PMC1731825

[19] Eibner C Sturm R. US-based indices of area-level deprivation: results from HealthCare for communities. Soc Sci Med. (2006) 62:348–59. doi: 10.1016/j.socscimed.2005.06.017 16039764

[20] Koimtzis G Geropoulos G Chalklin C Karniadakis I Szabo L Ilham MA. The influence of socioeconomic deprivation on outcomes in transplant patients infected with SARS-CoV-2 in Wales. Clin Transpl. (2024) 38:e15245. doi: 10.1111/ctr.15245, PMID: 38289884

[21] Rollings KA Noppert GA Griggs JJ Melendez RA Clarke PJ. Comparison of two area-level socioeconomic deprivation indices: implications for public health research, practice, and policy. PLoS One. (2023) 18:e0292281. doi: 10.1371/journal.pone.0292281, PMID: 37797080 PMC10553799

[22] Guillaume E Pornet C Dejardin O Launay L Lillini R Vercelli M. Development of a cross-cultural deprivation index in five European countries. J Epidemiol Community Health. (2016) 70:493–9. doi: 10.1136/jech-2015-205729, PMID: 26659762 PMC4853548

[23] Jaffiol C Thomas F Spira A Pannier B Danchin N. Prediabetes and deprivation: a couple at high risk of diabetes. Rev Epidemiol Sante Publ. (2021) 69:361–5. doi: 10.1016/j.respe.2021.04.139, PMID: 34053795

[24] Payne NWS Brown KF Delon C Kotrotsios Y Soerjomataram I Shelton J. Socio-economic deprivation and cancer incidence in England: quantifying the role of smoking. PLoS One. (2022) 17:e0272202. doi: 10.1371/journal.pone.0272202, PMID: 36129905 PMC9491592

[25] Qi X Jia Y Pan C Li C Wen Y Hao J. Index of multiple deprivation contributed to common psychiatric disorders: a systematic review and comprehensive analysis. Neurosci Biobehav Rev. (2022) 140:104806. doi: 10.1016/j.neubiorev.2022.104806, PMID: 35926729

[26] Moussaoui S Chauvin P Ibanez G Soler M Nael V Morgand C. Construction and validation of an individual deprivation index: a study based on a representative cohort of the Paris metropolitan area. J Urban Health. (2022) 99:1170–82. doi: 10.1007/s11524-022-00648-0, PMID: 35653078 PMC9161768

[27] Tricco AC Lillie E Zarin W O'Brien KK Colquhoun H Levac D. PRISMA extension for scoping reviews (PRISMA-ScR): checklist and explanation. Ann Intern Med. (2018) 169:467–73. doi: 10.7326/M18-0850, PMID: 30178033

[28] Terwee CB Bot SD de Boer MR van der Windt DA Knol DL Dekker J. Quality criteria were proposed for measurement properties of health status questionnaires. J Clin Epidemiol. (2007) 60:34–42. doi: 10.1016/j.jclinepi.2006.03.012 17161752

[29] Fouchard A Bréchat PH Castiel D Pascal J Sass C Lebas J. Caractéristiques métrologiques et comparaison de trois outils de repérage de la précarité sociale dans une permanence d’accès aux soins de santé hospitalière à Paris. Rev Epidemiol Sante Publ. (2014) 62:237–47. doi: 10.1016/j.respe.2014.04.004 25026886

[30] Page MJ McKenzie JE Bossuyt PM Boutron I Hoffmann TC Mulrow CD. The PRISMA 2020 statement: an updated guideline for reporting systematic reviews. BMJ. (2021) 372:n71. doi: 10.1136/bmj.n71 PMC8005924 33782057

[31] Besnier M Castiel D Bréchat PH Grenouilleau MC Rymer R Barranger E. Parturientes accouchant par voie basse, handicaps sociaux et durée de séjour: étude pilote au groupe hospitalier Lariboisière-Fernand-Widal de Paris. Gynecol Obstet Fertil. (2009) 37:131–9. doi: 10.1016/j.gyobfe.2008.08.017, PMID: 19200763

[32] Eroğlu S. Developing an index of deprivation which integrates objective and subjective dimensions: extending the work of Townsend, Mack and Lansley, and Halleröd. Soc Indic Res. (2007) 80:493–510. doi: 10.1007/s11205-006-0004-0

[33] Guio A-C Marlier E Gordon D Fahmy E Nandy S Pomati M. Improving the measurement of material deprivation at the European Union level. J Eur Soc Policy. (2016) 26:219–333. doi: 10.1177/0958928716642947

[34] Hofbauer LM Rodriguez FS. Association of social deprivation with cognitive status and decline in older adults. Int J Geriatr Psychiatry. (2021) 36:1085–94. doi: 10.1002/gps.5555, PMID: 33860548

[35] Labbe E Blanquet M Gerbaud L Poirier G Sass C Vendittelli F. A new reliable index to measure individual deprivation: the EPICES score. Eur J Pub Health. (2015) 25:604–9. doi: 10.1093/eurpub/cku231 25624273

[36] Matern RV Mendelson M Oliphant M (eds.) (2009). Developing a Deprivation Index: The Research Process. (Daily Bread Food Bank and the Caledon Institute of Social Policy).

[37] Andrew MK Mitnitski AB Rockwood K. Social vulnerability, frailty and mortality in elderly people. PLoS One. (2008) 3:e2232. doi: 10.1371/journal.pone.0002232, PMID: 18493324 PMC2375054

[38] Gu D Yang F Sautter J. Socioeconomic status as a moderator between frailty and mortality at old ages. BMC Geriatr. (2016) 16:151. doi: 10.1186/s12877-016-0322-2, PMID: 27506542 PMC4979157

[39] Møller JK la Cour K Pilegaard MS Möller S Jarlbaek L. Identification of socially vulnerable cancer patients—development of a register-based index (rSVI). Support Care Cancer. (2022) 30:5277–87. doi: 10.1007/s00520-022-06937-3, PMID: 35275294

[40] Pascal J Abbey-Huguenin H Agard C Asseray N Billaud É Baron D. Élaboration d’un outil de repérage des usagers en situation de vulnérabilité sociale consultant à l’hôpital. Presse Med. (2004) 33:710–5. doi: 10.1016/S0755-4982(04)98726-X 15257227

[41] Whelan CT Maître B. Vulnerability and multiple deprivation perspectives on economic exclusion in Europe: a latent class analysis. Eur Soc. (2005) 7:423–50. doi: 10.1080/14616690500194050

[42] O’Donnell P O’Donovan D Elmusharaf K. Measuring social exclusion in healthcare settings: a scoping review. Int J Equity Health. (2018) 17:15. doi: 10.1186/s12939-018-0732-1, PMID: 29391016 PMC5796599

[43] van Bergen APL Wolf JRLM Badou M de Wilde-Schutten K Ijzelenberg W Schreurs H. The association between social exclusion or inclusion and health in EU and OECD countries: a systematic review. Eur J Pub Health. (2018) 29:575–82. doi: 10.1093/eurpub/cky143 PMC6532832 30084924

[44] Darin-Mattsson A Fors S Kåreholt I. Different indicators of socioeconomic status and their relative importance as determinants of health in old age. Int J Equity Health. (2017) 16:173. doi: 10.1186/s12939-017-0670-3, PMID: 28950875 PMC5615765

[45] Wheeler DC Czarnota J Jones RM. Estimating an area-level socioeconomic status index and its association with colonoscopy screening adherence. PLoS One. (2017) 12:e0179272. doi: 10.1371/journal.pone.0179272, PMID: 28594927 PMC5464664

[46] Fukukawa Y Tsuboi S Niino N Ando F Kosugi S Shimokata H. Effects of social support and self-esteem on depressive symptoms in Japanese middle-aged and elderly people. J Epidemiol. (2000) 10:S63–9. doi: 10.2188/jea.10.1sup_63, PMID: 10835830

[47] Thomas PA Liu H Umberson D. Family relationships and well-being. Innov Aging. (2017) 1:igx 025. doi: 10.1093/geroni/igx025, PMID: 29795792 PMC5954612

[48] Uchino BN Way BM. Integrative pathways linking close family ties to health: a neurochemical perspective. Am Psychol. (2017) 72:590–600. doi: 10.1037/amp0000049, PMID: 28880105

[49] Umberson D Montez JK. Social relationships and health: a flashpoint for health policy. J Health Soc Behav. (2010) 51:S54–66. doi: 10.1177/0022146510383501, PMID: 20943583 PMC3150158

[50] Thoits PA. Stress and health: major findings and policy implications. J Health Soc Behav. (2010) 51:S41–53. doi: 10.1177/0022146510383499, PMID: 20943582

[51] Everson-Rose SA Lewis TT. Psychosocial factors and cardiovascular diseases. Annu Rev Public Health. (2005) 26:469–500. doi: 10.1146/annurev.publhealth.26.021304.144542 15760298

[52] Berkman LF Syme SL. Social networks, host resistance, and mortality: a nine-year follow-up study of Alameda County residents. Am J Epidemiol. (1979) 109:186–204. doi: 10.1093/oxfordjournals.aje.a112674, PMID: 425958

[53] Mathur MB Fox MP. Toward open and reproducible epidemiology. Am J Epidemiol. (2023) 192:658–64. doi: 10.1093/aje/kwad007, PMID: 36627249 PMC10089067

[54] Baumstarck K Boyer L Auquier P. The role of stable housing as a determinant of poverty-related quality of life in vulnerable individuals. Int J Qual Health Care. (2015) 27:356–60. doi: 10.1093/intqhc/mzv052, PMID: 26245324

[55] Chatelard S Bodenmann P Vaucher P Herzig L Bischoff T Burnand B. General practitioners can evaluate the material, social and health dimensions of patient social status. PLoS One. (2014) 9:e84828. doi: 10.1371/journal.pone.0084828, PMID: 24454752 PMC3893170

[56] Jatrana S. Gender differences in self-reported health and psychological distress among New Zealand adults. Demogr Res. (2021) 45:693–726. doi: 10.4054/DemRes.2021.45.21

[57] Leiser S Déruaz-Luyet A N’Goran AA Pasquier J Streit S Neuner-Jehle S. Determinants associated with deprivation in multimorbid patients in primary care—a cross-sectional study in Switzerland. PLoS One. (2017) 12:e0181534. doi: 10.1371/journal.pone.0181534, PMID: 28738070 PMC5524289

[58] Muller D Paine S Wu LJ Signal TL. 0356 associations between ethnicity, socioeconomic deprivation and preschoolers’ sleep health in Aotearoa/New Zealand. Sleep. (2020) 43:A136-A. doi: 10.1093/sleep/zsaa056.353 31302070

[59] Pons A Whalley G Coffey S. A070 socioeconomic deprivation is associated with worse quality of life in patients undergoing ischaemic stress testing. Heart Lung Circul. (2020) 29:S30. doi: 10.1016/j.hlc.2020.05.075

[60] Tatoli R Lampignano L Donghia R Castellana F Zupo R Bortone I. Dietary customs and social deprivation in an aging population from southern Italy: a machine learning approach. Front Nutr. (2022) 9:811076. doi: 10.3389/fnut.2022.811076 35340551 PMC8942783

[61] Thayer ZM Kuzawa CW. Early origins of health disparities: material deprivation predicts maternal evening cortisol in pregnancy and offspring cortisol reactivity in the first few weeks of life. Am J Hum Biol. (2014) 26:723–30. doi: 10.1002/ajhb.22532, PMID: 24599586

[62] Andrew MK Mitnitski A Kirkland SA Rockwood K. The impact of social vulnerability on the survival of the fittest older adults. Age Ageing. (2012) 41:161–5. doi: 10.1093/ageing/afr176, PMID: 22287038

[63] Andrew MK Rockwood K. Social vulnerability predicts cognitive decline in a prospective cohort of older Canadians. Alzheimers Dement. (2010) 6:319–325.e1. doi: 10.1016/j.jalz.2009.11.001, PMID: 20630414

[64] Armstrong JJ Andrew MK Mitnitski A Launer LJ White LR Rockwood K. Social vulnerability and survival across levels of frailty in the Honolulu-Asia aging study. Age Ageing. (2015) 44:709–12. doi: 10.1093/ageing/afv016, PMID: 25758407 PMC4476846

[65] Flanagan BE Gregory EW Hallisey EJ Heitgerd JL Lewis B. A social vulnerability index for disaster management. J Homeland Secur Emerg Manag. (2011) 8:1–22. doi: 10.2202/1547-7355.1792

[66] McCartney G Hoggett R Walsh D Lee D. How well do area-based deprivation indices identify income-and employment-deprived individuals across Great Britain today? Public Health. (2023) 217:22–5. doi: 10.1016/j.puhe.2023.01.020, PMID: 36841035

