# Supplementary material for: Individual-based socioeconomic vulnerability and deprivation indices: a scoping review
Source: Front Public Health. 2024 Aug 14;12:1403723. doi: 10.3389/fpubh.2024.1403723 (PMC11349641; doi:10.3389/fpubh.2024.1403723)
Supplement: Supplementary file 2 [file Table_2.DOCX]

**Table S2**: Variables found in one deprivation or vulnerability index

| **FREQUENCY** | **VARIABLE** |
| --- | --- |
| 1 TIME | Place of residence (urban/rural)  Help in daily life in need from someone close to the person Moral/emotional support in need from someone close to the person  Help in the form of clothes or money from a community organization  Buying cheap food  Meeting with a social worker (welfare worker, educator)  Social participation  Able to buy modest presents for family/friends at least once per year.  Are there people in your entourage on whom you can count to accommodate you for a few days if necessary  Able to get dental care if needed  Able to replace or repair broken electrical goods  Number of furniture and electrical appliances  Individual subscription to urban utilities (water, phone, electricity)  Cannot face unexpected financial expenses  Having appropriate clothes for job interviews  Having a home or apartment free of pests  Number of children in compulsory or higher education  Difficulties paying for medication or medical exams  Need to borrow money for daily expense  Perceived neighborhood safety  Imprisonment  Do you read a newspaper or books  Difficulties with telephone calls  Self-rated writing skills  Self-rated reading skills  Voluntary or charity work  Provided help to family, friends or neighbors  Attended educational or training course  Taken part in religious organization  Taken part in a political or community related organization  How satisfied are you with your life in general?  I feel left out of things  I feel that life is full of opportunities  I felt people were unfriendly  I felt that people disliked me  I am always satisfied with balance between what I’ve given my partner & what I’ve received in return  I’ve always received adequate appreciation for providing help in my family  In my current major activity, I’m always satisfied with rewards received for efforts  I have been seriously disappointed or hurt by someone to whom i gave my trust  How often do you have conflict with parents  How often do you have conflict with parents-in-law  How often do you have conflict with partner/spouse  How often do you have conflict with children  How often do you have conflict with other family members |
